# Partial Body Weight-Supported Treadmill Training in Spinocerebellar Ataxia

**DOI:** 10.1155/2018/7172686

**Published:** 2018-01-08

**Authors:** Laura Alice Santos de Oliveira, Camilla Polonini Martins, Carlos Henrique Ramos Horsczaruk, Débora Cristina Lima da Silva, Luiz Felipe Vasconcellos, Agnaldo José Lopes, Míriam Raquel Meira Mainenti, Erika de Carvalho Rodrigues

**Affiliations:** ^1^Post-Graduation Program in Rehabilitation Sciences, Augusto Motta University Center (UNISUAM), Rio de Janeiro, RJ, Brazil; ^2^School of Physiotherapy, Federal Institute of Rio de Janeiro, Rio de Janeiro, RJ, Brazil; ^3^Institute of Neurology Deolindo Couto, Federal University of Rio de Janeiro (UFRJ), Rio de Janeiro, RJ, Brazil; ^4^Physical Education College of the Brazilian Army (EsEFEx), Rio de Janeiro, RJ, Brazil; ^5^D'Or Institute for Research and Education (IDOR), Rio de Janeiro, RJ, Brazil

## Abstract

**Background and Purpose:**

The motor impairments related to gait and balance have a huge impact on the life of individuals with spinocerebellar ataxia (SCA). Here, the aim was to assess the possibility of retraining gait, improving cardiopulmonary capacity, and challenging balance during gait in SCA using a partial body weight support (BWS) and a treadmill. Also, the effects of this training over functionality and quality of life were investigated.

**Methods:**

Eight SCA patients were engaged in the first stage of the study that focused on gait training and cardiovascular conditioning. From those, five took part in a second stage of the study centered on dynamic balance training during gait. The first and second stages lasted 8 and 10 weeks, respectively, both comprising sessions of 50 min (2 times per week).

**Results:**

The results showed that gait training using partial BWS significantly increased gait performance, treadmill inclination, duration of exercise, and cardiopulmonary capacity in individuals with SCA. After the second stage, balance improvements were also found.

**Conclusion:**

Combining gait training and challenging tasks to the postural control system in SCA individuals is viable, well tolerated by patients with SCA, and resulted in changes in capacity for walking and balance.

## 1. Introduction

Spinocerebellar ataxia (SCA) comprises a family of autosomal dominant inherited disorders that result from progressive degeneration of the cerebellum and its associated systems [1]. Besides cerebellar deterioration, SCA is often accompanied by degeneration of other sites of the nervous system, leading to noncerebellar signs such as pyramidal and extrapyramidal losses, which are uncommon in ataxia of other etiologies and that can worsen the impairments of people with SCA [2].

Among the motor deficits prompted by SCA, those related to gait and balance are the most common [3]. Gait in SCA is usually described as uncoordinated, unsteady, wide-based, and highly variable [4–6]. In turn, balance abnormalities in SCA are characterized by an increased postural sway and poor balance control during both static and dynamic tasks [7]. It is noteworthy that both the balance and gait impairments in SCA are strongly associated with an increased number of fall episodes [8, 9] and can favor physical inactivity, adversely affecting cardiorespiratory fitness [10]. Together, these problems can impair mobility, deteriorate general health, and yield physical and social consequences for these individuals [7].

Despite the huge recent advances in neurogenetic research, an effective pharmacological approach to face this condition is still unknown [11, 12]. Indeed, with the exception of a few kinds of hereditary ataxia (e.g., Niemann–Pick disease type C, cerebrotendinous xanthomatosis, coenzyme Q-10 responsive ataxia, or ataxia with vitamin E deficiency), no specific treatments exist for hereditary ataxia, including SCA (for a review, see Jayadev and Bird [13]). In this context, rehabilitation strategies could represent an alternative to improve the physical condition and to reduce the impairments of these individuals. But, sadly, clinical trials testing the effects of physical therapy approaches in ataxia are scarce and the few existing studies include cerebellar ataxias of different etiologies, beyond SCA (for a review, see Martins et al. [14]) [15, 16]. As the natural course and prognosis are different between SCA and cerebellar ataxias of other etiologies it could be artificial to generalize the results from the available clinical trials to the SCA population, especially considering the peculiarity of the progressive degeneration found in SCA. In this context, it is relevant to advance strategies of rehabilitation that could benefit SCA individuals.

One strategy suggested to improve gait in ataxic individuals is to attach weights to their ankles or trunk. Although widely used in everyday physiotherapy practice, there is no consensus about the efficacy of this compensatory approach [17–20]. More recently it was proposed that alleviating the weight of cerebellar ataxic individuals during gait training could represent a perspective to improve this activity [21–23]. The neuromuscular impairments from ataxia could favor physical inactivity that can lead to additional neuromuscular and cardiopulmonary disturbances affecting overall functionality and quality of life [10, 24].

One alternative that has been successfully employed to improve gait performance in people with motor impairments is partial body weight support associated with gait training on a treadmill (PBWSTT). It consists of using an overhead harness to support a percentage of body weight while walking. The advantages of using PBWSTT are to provide task-oriented training; to allow several repetitions of a supervised gait pattern with an almost null fall risk; to enable an increasing pace of effort and postural control demands; and to make possible improvements of cardiopulmonary capacity [25–28]. Some case reports describe successful use of PBWSTT for gait and balance problems of non-SCA individuals [21–23]. Although providing promising results the feasibility and general effects of this strategy have not yet been tested in SCA individuals.

Studies based on dynamic balance training of SCA individuals are less scarce but also include cerebellar ataxia of other etiologies [16, 17]. Recently, our group tested the effectiveness of a modified version of an exercise program proposed by Ilg et al. [16] to improve coordination and balance [24]. Exercises for static and dynamic balance were applied exclusively to people with SCA and seem to be safe, with improvements in fall risk and balance in the sample of individuals studied [24]. Likewise, challenging balance during dynamic tasks such as gait training in SCA individuals will probably have an important impact on functionality since walking is an everyday activity that allows independence and autonomy in various other activities and individuals' social roles.

Until now, there has been no consensus in the literature about the best way to cope with the gait and balance problems presented by people with SCA. Therefore, the aim of the present pilot study was to assess the possibility of retraining gait, recovering/improving cardiopulmonary capacity, and challenging balance during gait in people with SCA using a partial body weight support device associated with a treadmill and to observe its effects on functionality and quality of life.

## 2. Materials and Methods

This was a pretest-posttest quasi-experimental open-label uncontrolled study design. It began with a set of assessments, followed by a PBWSTT protocol performed in 2 stages (gait/conditioning and dynamic balance training) followed by another set of assessments at the end of each stage. This study was approved by the local ethics committee (process number 17754813.0.0000.5235) and was carried out according to the Declaration of Helsinki. Written consent was obtained from all participants.

### 2.1. Participants

Twenty-five individuals with ambulatory movement disorders from a local neurological hospital that had positive genetic testing for any SCA subtype were invited to participate. Ten refused participation. Fifteen accepted the invitation and underwent an interview to search for eligibility criteria. Inclusion criteria were having received a diagnosis of SCA from a neurologist; answering “no” to all questions of the PAR-Q (a questionnaire that determines the possible risk of exercising for an individual based on the answers to specific health history questions [29]); and being able to walk 10 meters with or without a device. Exclusion criteria included being at “stage 0” (no gait difficulties) of ataxia disease [30] and the presence of vertigo, hypertension, postural hypotension, heart or coronary disease, epilepsy, or orthopedic problems that could limit gait. Five participants were excluded because of these criteria, 1 who was not able to ambulate and 4 because they were at “stage 0” of ataxia. Thus, 10 participants matched the exclusion/inclusion criteria and began the protocol of intervention. During the study, 2 participants dropped out of the protocol due to personal problems. Eight individuals (5 male) aged 27 to 58 (43 ± 11 years) participated in this pilot study. All of them gave informed written consent.  Table 1 summarizes the personal characteristics of the participants.

### 2.2. Intervention Procedures

Firstly, all participants underwent 2 sessions of assessment, with 1 week of interval between them. One aimed to evaluate cardiopulmonary capacity during exercise and the other functionality and quality of life (see below). Immediately after the last evaluation, participants started the PBWSTT protocol. Before and after each session of PBWSTT, participants had their blood pressure measured. During the session they also had their heart rate monitored. PBWSTT was implemented using a Biodex 500 harness (Biodex; Shirley, New York) with the capacity to lift up to 82 kg. Each participant was attached to the harness apparatus with a set of straps and fittings, by which he/she was fastened to the PBWSTT device. The support vest was secured tightly around the lower trunk of the participant, allowing hip flexion and extension. After that, the participant was invited to climb onto the treadmill (Ecafix EG 700.2). Participants started the program with 30% of their body weight alleviated by the device.

In the first stage of the PBWSTT program (gait/conditioning training), the main goals were (i) to progressively reduce body support to zero and (ii) to progressively increase velocity to the “maximal” for each individual. In this context, “maximal” was considered the highest speed the patient could achieve during the treadmill training without running. In the second stage (dynamic balance training), the main goal was challenging balance during gait. The first and second stages lasted 8 and 10 weeks each, respectively, both comprising a 50 min duration session with a frequency of 2 times per week.

As mentioned, in the gait/conditioning training stage, participants started walking on a treadmill with 30% of their body weight alleviated by the device. In this stage the participants were free to hold onto the handrails. The sessions were divided as follows: the first 10 min was dedicated to cardiovascular system warm-up by increasing the speed until reaching the maximal velocity (as described above) for that individual without running or losing coordination. For the next 30 min, the participant walked at this maximal speed. Their perceived effort was monitored every 5 min (Borg modified scale—0 to 10 [31]). Finally, in the course of the last 10 min, the speed was gradually decreased to zero to allow the heart rate to slow down and breathing to return to normal levels. The maximal speed during treadmill training was increased individually over the course of the 8 weeks, varying depending on participant ability and capacity. Additionally, the percentage of corporeal weight supported by the device was gradually decreased until the participant was able to walk attached to the support but without any body weight alleviated. At the end of the 8 weeks of this first stage, cardiopulmonary capacity, functional capacity, and quality of life of the participants were evaluated again.

**Figure 1:**
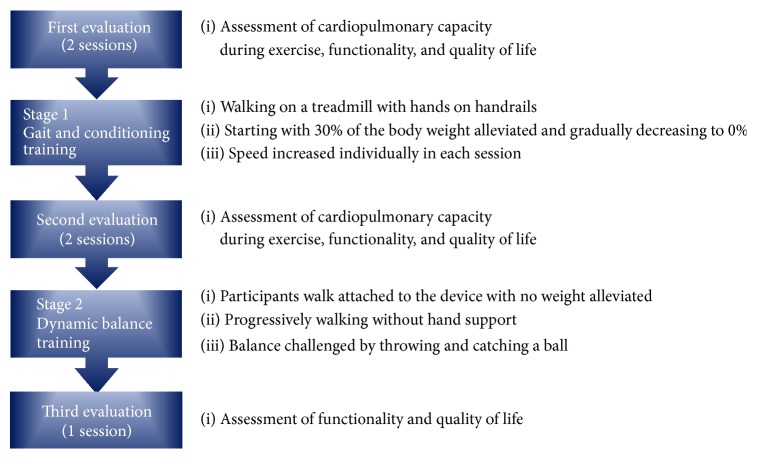
The experimental protocol.

After the evaluation, the dynamic balance training stage began. In this second stage, participants walked attached to the body weight support device without any weight alleviated, at the maximal velocity that they had reached at the end of stage 1. The first and last 10 min of training were also dedicated to warm-up and cool-down of participants. During the middle 30 min, participants were stimulated to progressively walk without any hand support. About 2 weeks after that, the participant's balance started to be challenged by throwing and catching a ball to the individual while walking on the treadmill, at intervals of 5 min, intercalated by 1 min of rest. At the end of this stage, the functional capacity and quality of life, but not cardiopulmonary capacity, of the participants were evaluated again.  Figure 1 summarizes all procedures.

### 2.3. Outcome Measures

Physiotherapists and a physician expert in the instruments employed in this study performed all evaluations.

#### 2.3.1. Cardiopulmonary Performance

The cardiopulmonary performance during exercise was assessed through cardiopulmonary exercise testing (CPET). Respiratory gas exchange was sampled from a mouthpiece connected to a medium flow pneumotachograph (Medgraphics, Minnesota, USA) and a gas analyzer (VO2000, Medgraphics, Minnesota, USA), calibrated before each test with gas standards of known concentrations [oxygen (O_2_) = 12.0%; carbon dioxide (CO_2_) = 5.3%]. The ventilation flow and O_2_ and CO_2_ expired fractions were measured breath by breath. A nose clip was used to avoid gas escape. The CPET was performed on a treadmill (FE 700.2, Ecafix, São Paulo, Brazil) using a modified version of the Naughton protocol [32]. Participants were instructed to hold their hands on the treadmill bars during the entire test to avoid falls. Before the test, the participants underwent a familiarization period of about 1 min of walking on the treadmill (low velocity, no inclination). After that, the test began with an initial speed of 1.6 km/h with one increment to 3.2 km/h after 3 min. Increments were performed only in graduation, with a rise of 3.5° every 3 min. All participants were informed about the test interruption criteria: chest pain, systolic blood pressure (SBP) > 220 mmHg, diastolic blood pressure (DBP) > 115 mmHg, a drop in SBP despite an increase in workload, dizziness, physical manifestations of extreme fatigue, ECG changes, and the subject's request to stop and when the maximal grade was achieved [29]. The Borg perception effort scale was used to check the degree of perceived effort at every step of the test [31].

The relative oxygen consumption (VO_2_; mL/kg/min) and minute ventilation (VE; L/min) variables were considered at the peak of the exercise. The peak VO_2_ was considered as the highest VO_2_ reached in the final minute of the effort. Other variables analyzed included the duration of the CPET and the maximum inclination achieved on the treadmill during this test.

#### 2.3.2. Functional Capacity and Quality of Life

Functional capacity was assessed in respect of balance, gait, and severity of ataxia. Balance was assessed with the Brazilian Portuguese validated version of the Berg balance scale (BBS) [33], in which scores range from 0 to 56 [34]. The higher the score in the BBS, the better the postural control. Chiu et al. [35] suggested that BBS scores equal to or less than 45 points indicate an increased fall risk. Here, the same criterion was used. The participants' ability to respond to demands during walking was assessed with the dynamic gait index (DGI), in which scores range from 0 (high risk of falls) to 24 (low risk of fall) [36]. Scores equal to or less than 19 are associated with an increased risk of falling. To assess the severity of ataxia we used both the scale for the assessment and rating of ataxia (SARA) score, a standardized clinical measure of neurologic manifestations of cerebellar ataxia in which scores range from 0 (no ataxia symptoms) to 40 (most severe ataxia) [37], and the brief ataxia rating scale (BARS), with a total score of 30 points (most severe ataxia) [38]. Quality of life was assessed by means of the Katz index of independence in activities of daily living (Katz ADL), an instrument to assess functional status by measuring an individual's ability to perform activities of daily living independently, in which scores vary from 6 (patient independent) to 0 (patient very dependent) [39].

### 2.4. Statistics

Results were expressed as the median, minimum and maximum value ranges. Given the nonnormal distribution of the data (Kolmogorov–Smirnov), a Wilcoxon matched-pairs test was applied for comparisons of outcome measures of functional capacity, quality of life, and cardiopulmonary capacity obtained before and after gait/conditioning training stages. A Wilcoxon matched-pairs test was also used for comparisons between functional capacity and quality of life before and after the dynamic balance training. A significance level of 5% was used. The statistical analyses were performed using Statistica 7 software.

## 3. Results

**Figure 2:**
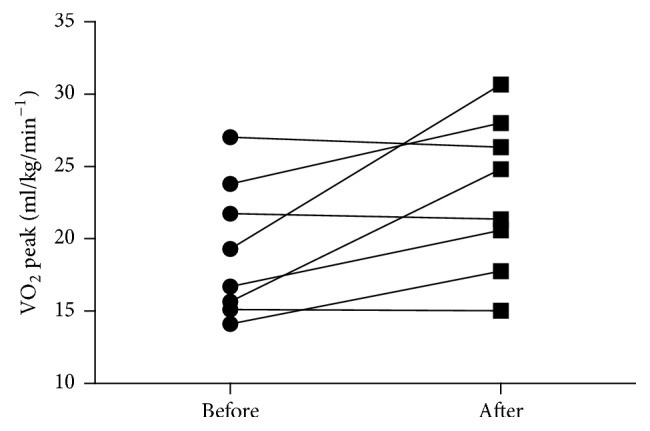
VO_2_ Peak values for each participant before and after the gait/conditioning stage.

Eight SCA individuals participated in the first stage (gait/conditioning) of this study. From those, 5 also took part in the second stage (dynamic balance training). After the gait/conditioning stage the participants showed significant improvements in CPET duration (*P* < 0.01) and the maximal treadmill inclination achieved during the test (*P* < 0.01; see Table 2). The VE Peak (*P* = 0.050) and VO_2_ Peak did not show statistical significance (*P* = 0.093) after intervention. Despite the nonsignificance, however, from Figure 2 it is possible to see that a few SCA individuals benefit from training, as 5 individuals presented an increment while 3 show a slight decrement of VO_2_ Peak.

The gait/conditioning training also had an impact on postural demands during walking, as observed in the DGI scores compared with before the intervention (Table 2).

The dynamic balance training stage brought significant improvements in balance as measured by the BBS scores when this parameter was compared before and after the intervention (Table 3), but without further changes in DGI scores.

SARA and Katz ADL scores did not show any changes after the intervention (Tables 2 and 3).

## 4. Discussion

This study is the first, to our knowledge, to investigate the feasibility and consequences of the association of gait training and balance challenges using PBWSTT on functionality, cardiopulmonary capacity, and quality of life in SCA individuals. The results demonstrated that the training was feasible and well tolerated by people with SCA. Trends of improvements were found after the gait/conditioning training using PBWSTT in the capacity for walking by increasing the gait performance and the cardiopulmonary capacity of the sample of individuals studied. The dynamic balance training also brought statistically significant improvements in balance.

At the end of the gait/conditioning training stage, as expected, improvements were found in gait as measured by DGI. SCA participants were also capable of walking with higher inclination of the treadmill for longer periods of time in the CPET. Probably, several mechanisms play a role in these improvements. The use of task-oriented training and increasing the pace of effort may have been relevant [25–27]. Additionally, the majority of studies with PBWSTT attribute the gait improvements observed to changes in the central pattern generator in different conditions such as Parkinson's disease, spinal cord injury, and stroke (e.g., Wickelgren [40] and Miyai et al. [41]). Although there was no statistical difference between the VO_2_ Peak after and before the gait/conditioning training stage it was observed that, in 5 of 8 participants tested, there was a VO_2_ Peak increment. It was expected since treadmill training has already been associated with cardiopulmonary capacity improvement after stroke and in individuals with coronary artery disease [42, 43]. Moreover, the level of intensity of CPET increased for the group (as suggested by treadmill inclination and CPET duration increments), suggesting an increased correspondent effort during its execution. It may explain the absence of VO_2_ Peak and Borg improvement for some individuals.

As expected, after the dynamic balance training stage, an improvement of balance was observed as measured by BBS. In this stage the strategy was to challenge balance by throwing and catching a ball to the individual during treadmill training. Keeping the patient attached to the body weight device even without alleviating weight helped to avoid falls, providing the possibility of performing exercises that otherwise would be very dangerous to SCA individuals. In fact, it is already known that SCA is strongly associated with an increased number of fall episodes [8, 9]. In a one-year period, 73.6% of 228 SCA patients reported at least one fall; from these, 74% related a high rate of fall-related injuries [8]. Another study showed that, from 113 SCA patients that recorded their falls in a diary during one year, 84.1% reported at least one fall. One method to measure fall status can be through the BBS score [36]. Here the increment of BBS scores indicates a decrease in participants' fall risk, which is very relevant, due to the impact of falls on morbidity and mortality in this population [4]. It is already known that balance perturbations during walking improve balance in healthy older people and individuals who have suffered a stroke [44, 45]. A case study about a patient with progressive supranuclear palsy during walk training, balance perturbation, and step training using a PBWSTT also found improvements of gait and balance [46]. However, the exact mechanisms responsible for the balance improvement remain unclear. Given that SCA is a progressive disease that affects multiple central nervous system sites and the cerebellum that is essential in motor learning, this becomes even more challenging [2]. Finally, it is important to remember that, in this study, the individuals were not able to walk on the treadmill without holding onto handrails. So, without previous training of gait and improvement of cardiovascular conditioning, the dynamic balance training stage of exercises would not have been possible.

In respect of quality of life, the absence of change after the accomplishment of both stages of this study may be due to a ceiling effect. In fact, the major part of participants of this study was considered independent according to the Katz ADL index even during the first evaluation. Similarly, there are insufficient data to show that individuals after stroke, with Parkinson disease and spinal cord injury, improved their quality of life after PBWSTT [47–57].

The results relative to SARA scale were not as significant as expected. This may indicate that the progression of the disease had not changed in the course of this study, maybe due to its short duration.

The PBWSTT is largely used in the rehabilitation of patients with various neurological conditions beyond SCA, including Parkinson's disease, stroke, and spinal cord injury [47–57]. For example, in individuals with Parkinson's disease, PBWSTT was able to improve gait velocity, cadence, and step length, besides improving the weight distribution between lower limbs during walking [47–49]. Regarding the use of PBWSTT after stroke, there are reports of improvement related to the affected side: increases in step length, longer stance phase, larger swing phase, and a bigger distance traveled in the six-minute walk test [50–54]. Finally, the use of PBWSTT in spinal cord injury was being able to improve gait speed and backward gait speed, 6-minute walk distance, stride length. and mobility [55–57].

On the other hand, due to the novelty of the training protocol proposed here, contrasts with previous studies that involved cerebellar ataxic individuals are complex. Moreover, the studies available employed locomotor training strategies to improve gait in cerebellar ataxic individuals with non-SCA etiologies. For instance, Vaz et al. [21] reported promising evidence with treadmill training only, more strong in 1 of 2 participants with traumatic brain injury (TBI) and ataxic gait. There were gains in cadence, walking speed, step length, mobility, and balance. Two studies employed PBWSTT. Freund et al. [22] reported some gains in balance and gait in 1 ataxic individual by TBI that attained a program of exercises with PBWSTT. Cernak et al. [23] reported a single case of a 13-year-old girl who had a cerebellar/brainstem infarct and performed locomotor training with PBWSTT. She reached modified independence for transfers, supervision for walking, and minimal assistance for stairs after 6 months of intervention. Additionally, the studies proposing programs of exercise for gait and balance in ataxia did not include only people with SCA, highlighting the importance of the present study (for a review, see Martins et al. [14]) [15–17, 41].

The results of the present study are pioneering in suggesting that PBWSTT could be effective in improving both cardiopulmonary exercise capacity and balance during gait in SCA individuals, but some limitations need to be considered. The lack of a control group and the small sample size are the major constraints of the present study. We request that interpretation of the results should be done with caution, but it is important to keep in mind that SCA is considered a rare disease and its impairments make the mobility of outpatients difficult by limiting their participation in studies that require urban displacement. Another limitation is that although participants started the program with about 30% of their body weight alleviated by the device, these values are referred to a very heterogeneous sample of patients in terms of BMI, as it was indicated in the sample personal characteristics table. Finally, it would have been useful to quantify changes between pre- and posttraining by using instrumental tools (i.e., kinematic, kinetic, and EMG analysis) and range of motions, muscle behaviors, reaction forces, and internal moments specially because the experimental setup was based on gait and balance training.

We concluded that combining gait and conditioning training with dynamic balance training using a PBWSTT device is feasible and well tolerated by people with SCA. Additionally, it resulted in trends of improvement in capacity for walking and balance. A larger sample of SCA individuals is necessary to confirm these results by means of a randomized controlled clinical trial. Also, future studies would include more qualitative measures related to movement to evaluate the impact of this approach on gait.

## Figures and Tables

**Table 1:** Sample personal characteristics.

Participant	Sex	Age (yrs)	Weight (Kg)	Body height (m)	BMI (Kg/m^2^)	SCA type	Disease course (yrs)
1	F	32	84.2	1.68	29.83	2	12
2	M	27	56.3	1.76	18.18	3	4
3	M	31	54.9	1.71	18.78	3	7
4	M	58	64.2	1.70	22.21	3	8
5	M	44	113.2	1.73	37.82	3	6
6	F	47	43.5	1.66	15.79	7	4
7	F	52	53.9	1.66	19.56	3	7
8	M	54	85	1.72	28.57	3	12

F = female; M = male; Yrs = years; Kg = kilograms; m = meters; BMI = body mass index; Kg/m^2^ = weight in kilograms divided by height in meters squared; SCA = spinocerebellar ataxia.

**Table 2:** Outcome measures before and after the gait/conditioning stage.

Outcome measure	Before	After	*P* value
Duration			
Median/range	9.4/2.15–22.4	20.9/3.08–22.9	**0.04** ^*∗*^
Inclination %			
Median/range	5.25/0–10.5	21/0–21	**0.04** ^*∗*^
VE Peak L/min			
Median/range	26.6/17.3–54.1	32.1/23.3–56.5	0.05
Borg peak			
Median/range	17/15–20	15/11–20	0.55
DGI			
Median/range	13/1–21	16.5/8–24	**0.03** ^*∗*^
BBS			
Median/range	48.5/28–54	48/16–54	0.89
SARA			
Median/range	13.5/8–18.5	9/7–19.5	0.08

Values are median or range (minimum–maximum) of 8 individuals with SCA. VE Peak = minute ventilation for the peak of effort; DGI = dynamic gait index; BBS = Berg balance scale; SARA = scale for the assessment and rating of ataxia. *∗* = statistically significant in the Wilcoxon matched-pairs test.

**Table 3:** Outcome measures before and after the dynamic balance training stage.

Outcome measure	Before	After	*P* value
DGI			
Median/range	21/12–24	22/16–23	0.36
BBS			
Median/range	48/44–54	54/47–55	**0.04** ^*∗*^
SARA			
Median/range	7.5/7–12	9/5–13	0.90

Values are median and range (minimum–maximum) of 5 individuals with SCA. DGI = dynamic gait index; BBS = Berg balance scale; SARA = scale for the assessment and rating of ataxia. *∗* = statistically significant in the Wilcoxon matched-pairs test.
